# A 54-year-old Woman with Myelofibrosis and Massive Hemothorax Due to Primary Extramedullary Hematopoiesis of the Pleura

**DOI:** 10.7759/cureus.3675

**Published:** 2018-12-03

**Authors:** Michael Karass, Katherine Linder, Anup Agarwal, Alexandra Budhai, Yasmin Yusuf, Oleg Epelbaum

**Affiliations:** 1 Internal Medicine, Westchester Medical Center, Valhalla, USA; 2 Pathology, Westchester Medical Center, Valhalla, USA

**Keywords:** pleural, myelofibrosis, hematopoiesis, hemothorax, extramedullary, radiation

## Abstract

Extramedullary hematopoiesis, which represents ectopic blood cell production, is usually an incidental finding accompanying hematologic pathology. The liver and spleen are the most common sites of extramedullary hematopoiesis, but thoracic involvement is likewise observed. Pleural effusions in the setting of intrathoracic extramedullary hematopoiesis have been attributed to mechanical interactions between the pleural surface and neighboring paravertebral masses consisting of hematopoietic tissue. Rupture of these highly vascularized lesions into the adjacent pleural space has been the putative mechanism in cases complicated by hemothorax. Histologically proven instances of islets of extramedullary hematopoiesis occurring on the pleural surface itself are exceedingly rare. Our case of a patient with myelofibrosis and massive pleural effusion is only the third such example described in the literature and the second to result in a confirmed hemothorax requiring surgery. As expected, technetium-99m sulfur (Tc-99m sulfur) colloid scanning accurately localized sites of extramedullary hematopoiesis in our patient, and there was a salutary response to radiation therapy.

## Introduction

Extramedullary hematopoiesis (EMH) is a well-recognized feature of hematopoietic disorders such as myelofibrosis and thalassemia. Reported thoracic manifestations of EMH have included paravertebral mass lesions [1], diffuse alveolar hemorrhage [2], and pleural effusion [3]. There are numerous descriptions of hemothorax resulting from EMH [4-12], but in these cases, the bleeding was attributable to the rupture of the paravertebral foci of EMH into the adjacent pleural space. In contrast, the direct involvement of pleural tissue with EMH implants is an extremely rare finding confirmed histologically only thrice previously to our knowledge [13-15]. Only one of these three cases [14] was complicated by massive hemothorax. We report the second such occurrence.

## Case presentation

A 54-year-old woman with a 20-year history of myelofibrosis (MF) being treated with ruxolitinib and complicated by pancytopenia and massive splenomegaly initially presented to an outside hospital with progressive dyspnea and generalized weakness. Laboratory evaluation at that hospital revealed worsened anemia compared to the patient’s baseline. Chest imaging demonstrated a large left pleural effusion for which she underwent thoracentesis. She was eventually transferred to our institution for further management.

On presentation to our institution, the patient’s blood pressure was 131/85 mmHg, pulse 90 beats/min, and respiratory rate 18 breaths/min. She had a temperature of 98.1F and an oxygen saturation of 98% while breathing supplemental oxygen via a nasal cannula at 2 L/min. Mild respiratory distress was apparent. On cardiac examination, she had a grade 2/6 systolic ejection murmur. Lung examination was notable for decreased breath sounds at the left base with dullness to percussion. Other pertinent findings were pallor, palpable splenomegaly, and lower extremity edema.

**Figure 1:**
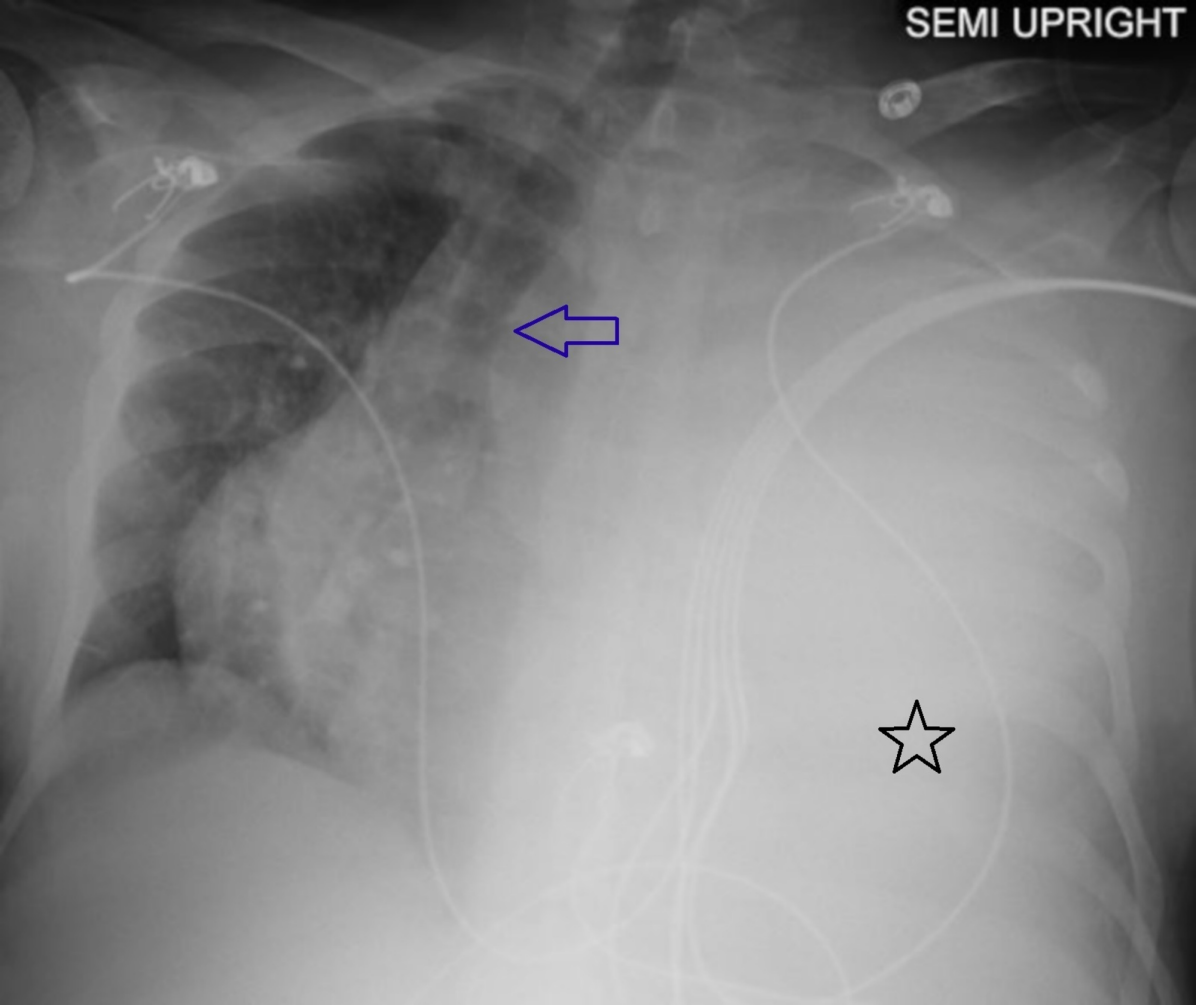
Left pleural effusion on chest X-ray Portable plain radiograph of the chest showing a massive left pleural effusion (black star) with contralateral displacement of trachea (blue arrow)

**Figure 2:**
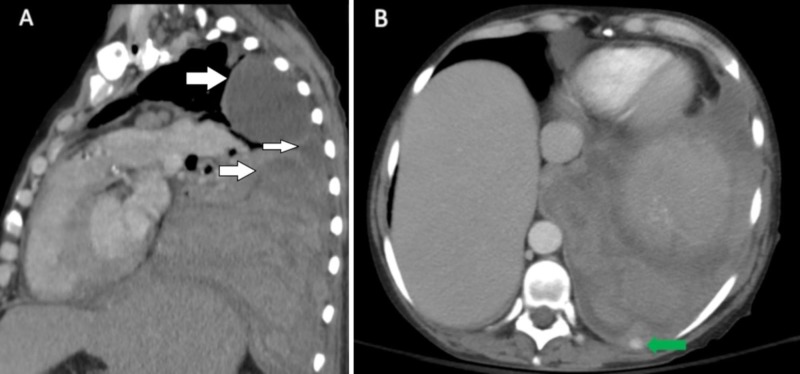
Multi-loculated pleural effusion and active bleeding on CT angiogram A) Coronal view from the computed tomography (CT) angiogram of the chest showing a multi-loculated pleural fluid collection in the left hemithorax (white arrows). B) Axial image from the CT angiogram showing extravasation of contrast (green arrow) compatible with active bleeding

**Figure 3:**
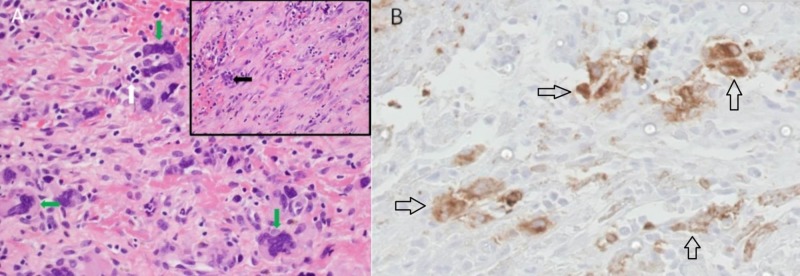
Pleural biopsy with megakaryocytes seen with hematoxylin & eosin and immunostaining A) Pleural biopsy section showing multiple megakaryocytes (green arrows) as well as an erythroid precursor (white arrow). Inset shows a different section containing a myeloid precursor (black arrow) (hematoxylin & eosin, original magnification x 400, inset x 100). B) Pleural tissue immuno-staining for CD61 (black arrows), which highlights megakaryocytes (original magnification x 400).

**Figure 4:**
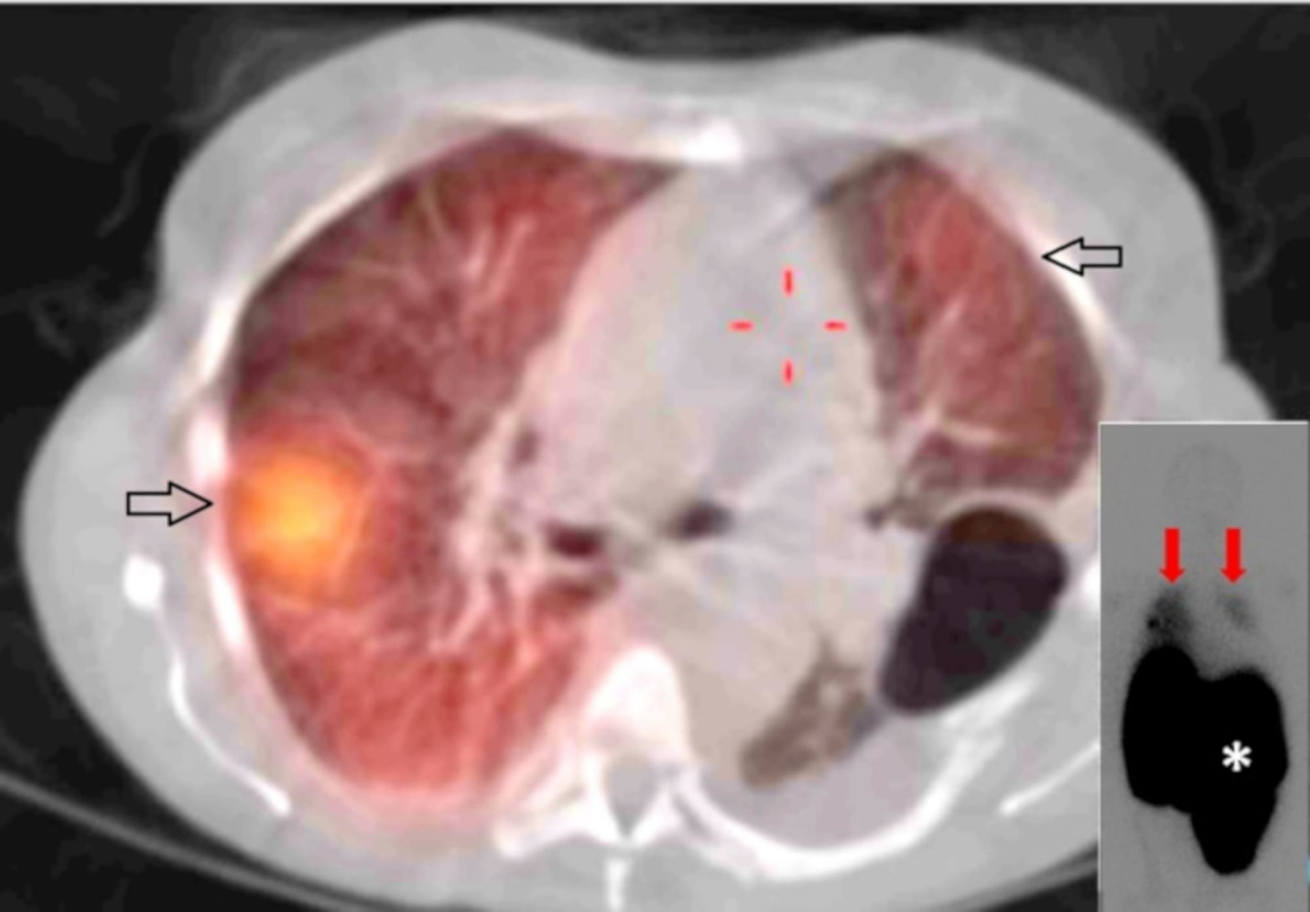
Increased uptake on SPECT/CT Technetium-99m sulfur (Tc-99m sulfur) colloid single photon emission computed tomography/CT (SPECT/CT) axial fusion image showing increased uptake in the thorax (black arrows). Planar scintigraphy images (inset lower right) likewise demonstrate increased uptake in both the right and left hemithorax (red arrows) while highlighting the massive splenomegaly characteristic of myelofibrosis (asterisk)

Abnormal laboratory values included a leukocytosis of 18.4 k/mm3 (normal range 4.8-10.8 k/mm3) with an increase in myeloid precursors, hemoglobin of 5.6 g/dl (normal range 12-16 g/dL), thrombocytopenia of 77 k/mm3 (normal range 160-410 k/mm3), and lactate dehydrogenase (LDH) of 882 U/L (normal range 125-220 U/L). Her coagulation parameters were unremarkable. Portable chest X-ray showed complete opacification of the left hemithorax with a rightward displacement of the trachea (Figure 1). Urgent placement of a left chest tube yielded grossly bloody pleural fluid consisting of a red blood cell (RBC) count of 1,415,000/mm3 and an LDH of 1555 U/L. The approximate pleural fluid hematocrit of 14% was >50% of serum hematocrit consistent with hemothorax. She received three units of packed RBCs over the ensuing two days but surgical intervention was not pursued. There was no evidence of malignancy by cytology or flow cytometry. The chest tube was removed on hospital Day 9, as there was no active drainage with an interval decrease of pleural effusion and stable serum hemoglobin. Over the subsequent two days, however, her serum hemoglobin again dropped from 8.0g/dL to 6.5g/dL. On hospital Day 11, computed tomography (CT) angiogram of the chest, abdomen, and pelvis was performed to localize a source of bleeding. The CT angiogram showed a large, complex fluid collection in the left pleural space measuring 11.6 x 9.6 cm in cross-section with a hyperdense focus compatible with active extravasation (Figure 2). The patient was taken to the operating room for urgent exploration. Approximately 2.5 liters of blood and blood clots were surgically evacuated. There was no evidence of a discrete bleeding source, but there was diffuse pleural oozing. Decortication of a thin fibrous peel was performed. She was extubated 24 hours postoperatively and transferred to the general medical floor after discontinuation of all chest tubes. A hematoxylin & eosin stained section of the patient’s pleura biopsied intraoperatively revealed maturing erythroid and myeloid cells with small clusters of erythroid and myeloid precursors (Figure 3A). Also present were atypical and dysplastic megakaryocytes that were highlighted by CD61 immunostaining (Figure 3B). The finding of trilineage hematopoiesis in a pleural sample is diagnostic of EMH at that location. Nuclear imaging with Technetium-99m sulfur (Tc-99m sulfur) colloid demonstrated diffusely increased uptake in the chest bilaterally, right greater than left, consistent with intrathoracic EMH (Figure 4). As expected, there was decreased activity within the bone marrow compatible with the patient's history of myelofibrosis.

## Discussion

EMH is the production of blood cell elements outside of the bone marrow. It can be a compensatory mechanism in bone marrow replacement pathology, such as MF, in hemoglobinopathies such as thalassemia and sickle cell disease, in red cell dyscrasias such as hereditary spherocytosis, and in many other hematologic conditions. The usual sites of EMH are the liver and spleen, but it has been encountered in virtually all organs [15]. Intrathoracic EMH is a rare but well-described finding; the most common locations are the paravertebral space within the posterior mediastinum, the ribs, and the lung parenchyma [16]. It is typically an incidental discovery in a patient with known hematopoietic pathology. Clinical and radiographic suspicion for this diagnosis can be supported by the increased uptake of technetium-99m sulfur colloid on nuclear scanning, which is a substance absorbed selectively by reticuloendothelial cells [17]. Tissue sampling of EMH sites is considered inadvisable due to the propensity of these densely vascularized structures for bleeding. Foci of EMH are exquisitely sensitive to radiation therapy (RT), which is the typical management strategy [18].

Within the already rare category of intrathoracic EMH, pleural involvement is exceedingly uncommon. The majority of existing reports describe patients with thalassemia who developed hemothorax [4-5,7-12] and, therefore, presented with dyspnea in contrast to uncomplicated cases of intrathoracic EMH, which tend to be asymptomatic. Based on information from reports with surgical or autopsy findings [4-5,8-12], pleural hemorrhage is most often the result of rupture of paravertebral, juxtapleural foci of EMH into the pleural space with resultant bleeding. This pathogenetic mechanism could be considered a secondary form of pleural EMH as opposed to the current case, which could be thought of as primary pleural EMH because lesions were actually detected in the pleural surface itself and were the likely source of bleeding. If one considered cases with identifiable pleural implants to be a separate entity, then this patient belongs to a unique category described in detail only twice previously [13-14]. The first such description [13] dates back to 1967 and reports a very similar patient: a 65-year-old woman with constitutional symptoms and a left pleural effusion. Thoracentesis was unsuccessful, so a pleural biopsy was performed, which revealed hematopoietic elements. Further evaluation led to the diagnosis of myelofibrosis, and the patient expired 18 months following her original presentation. At autopsy, the visceral pleural surface was found to be covered by nodules and plaques histologically composed of hematopoietic cells. The other case report [14] was published in 1993 and described a 73-year-old woman with known myelofibrosis who presented with dyspnea and a right pleural effusion. Initial thoracentesis yielded a serosanguinous exudative fluid not consistent with hemothorax. The pleural biopsy was non-diagnostic at that time. When the effusion recurred two weeks later, doxycycline pleurodesis was performed via tube thoracostomy, following which her symptoms and chest X-ray worsened. Emergent exploratory surgery resulted in the evacuation of approximately 2 liters of clotted blood. Decortication and pleural biopsy were performed, and the histological findings were consistent with EMH. She required radiation therapy and additional operative explorations to control the bleeding but subsequently succumbed to superimposed sepsis. Autopsy findings revealed multi-organ EMH.

The current patient is the third detailed description of primary EMH of the pleura, with the publication frequency of these three cases being approximately once every 25 years. Like the two before her, she is of female gender and has underlying myelofibrosis. Particularly instructive is the comparison with the more recent case from 1993, which also resulted in pleural hemorrhage. That patient initially presented with a pleural effusion that was not a hemothorax, and frank blood filled the pleural space only after subsequent chest tube placement and chemical pleurodesis. Although the results of the initial thoracentesis performed at the referring hospital in our patient are not available, it is reasonable to surmise based on the lack of urgent pleural intervention at that facility that the initial pleural fluid sample was not consistent with hemothorax. It is likely that the first discovery of hemothorax in our case occurred following pleural drain placement at our institution. This pattern suggests that pleural manipulation may be required to convert non-bleeding pleural EMH to a source of active hemorrhage. It appears that a subset of patients with pleural EMH carries asymptomatic implants, some present with pleural effusion that is not hemothorax, and yet others develop hemorrhage triggered by intervention. The possibility that many patients with hematopoietic disorders may harbor unrecognized foci of pleural EMH is illustrated by the 1967 case mentioned above as well as a patient included in a 2003 series from the Mayo Clinic [15] in which pleural EMH was incidentally discovered in the setting of chronic lymphocytic leukemia. Table 1 summarizes the three primary pleural EMH cases, including the current one, which has been described in detail.

**Table 1:** Summary of the three primary pleural extramedullary hematopoiesis cases described in detail in the literature MF = myelofibrosis, N/A = not applicable ^*^Patient died within a year of presentation ^#^Patient died during index hospitalization ^$^Patient alive three years after index admission

Citation/Year	Age/Gender	Underlying Disease	Hemothorax	Prior Pleural Intervention	Outcome
Anton et al (1967) [13]	65/F	MF	N	N/A	Death^*^
Kuperschmid et al (1993) [14]	73/F	MF	Y	Pleurodesis	Death^#^
Present Case (2018)	54/F	MF	Y	Drainage	Alive^$^

The patient underwent 200 cGy of RT over four days to the left pleura for pleural EMH based on findings on the nuclear scan, with a resolution of hemothorax, and was discharged to a rehabilitation facility. She is currently alive about three years after the event, making her the first survivor of massive hemothorax due to primary pleural EMH since the patient described in 1993 expired during the index hospitalization.

## Conclusions

EMH of the thorax is a well-described entity, but clinically overt involvement of the pleural space is rare. Hemothorax in the setting of EMH has been primarily reported in the setting of rupture of juxtapleural paravertebral EMH into the pleural space. Instances of hemothorax caused by primary pleural EMH are exceedingly uncommon, and bleeding in such cases may be precipitated by pleural intervention. Tc-99m sulfur colloid nuclear scanning can be used to confirm the suspicion of intrathoracic EMH, thus obviating the need to sample these richly vascularized lesions. EMH tissue is highly radiosensitive, so RT is the treatment of choice in symptomatic cases.
